# National disability-adjusted life years (DALYs) for 257 diseases and injuries in Ethiopia, 1990–2015: findings from the global burden of disease study 2015

**DOI:** 10.1186/s12963-017-0146-0

**Published:** 2017-07-21

**Authors:** Awoke Misganaw, Yohannes Adama Melaku, Gizachew Assefa Tessema, Amare Deribew, Kebede Deribe, Semaw Ferede Abera, Muluken Dessalegn, Yihunie Lakew, Tolesa Bekele, Tilahun N. Haregu, Azmeraw T. Amare, Molla Gedefaw, Mesoud Mohammed, Biruck Desalegn Yirsaw, Solomon Abrha Damtew, Tom Achoki, Jed Blore, Kristopher J. Krohn, Yibeltal Assefa, Mahlet Kifle, Mohsen Naghavi

**Affiliations:** 1 Institute for Health Metrics and Evaluation, University of Washington, Seattle, USA; 2 School of Public Health, University of Adelaide, Adelaide, Australia; 3 School of Public Health, College of Health Sciences, Mekelle University, Mek’ele, Ethiopia; 4 Department of Reproductive Health, Institute of Public Health, University of Gondar, Gondar, Ethiopia; 5 KEMRI-Wellcome Trust Research Programme, Kilifi, Kenya; 6 Nuffield Department of Clinical Medicine, University of Oxford, Oxford, UK; 7 St. Paul Millennium Medical College, Addis Ababa, Ethiopia; 8 Brighton and Sussex Medical School, Brighton, UK; 9 School of Public Health, Addis Ababa University, Addis Ababa, Ethiopia; 10 Institute for Biological Chemistry and Nutrition, University of Hohenheim, Stuttgart, Germany; 11 Amref Health Africa in Ethiopia, Addis Ababa, Ethiopia; 12 Ethiopian Public Health Association, Addis Ababa, Ethiopia; 13 Department of Public Health, College of Medicine and Health Sciences, Madda Walabu University, Bale Robe, Ethiopia; 14 Africa Population and Health Research Center, Nairobi, Kenya; 15 Federal Ministry of Health, Addis Ababa, Ethiopia; 16 College of Medicine and Health Sciences, Bahir Dar University, Bahir Dar, Ethiopia; 17 University of South Australia, Adelaide, Australia; 18 College of Health Sciences and Medicine, Wolayta Sodo University, Addis Ababa, Ethiopia; 19 School of Public Health, University of Queensland, St Lucia, Australia

**Keywords:** Disability-adjusted life years, Communicable disease, Maternal and neonatal disease, Nutritional deficiency disorders, Non-communicable diseases, Injuries, Ethiopia

## Abstract

**Background:**

Disability-adjusted life years (DALYs) provide a summary measure of health and can be a critical input to guide health systems, investments, and priority-setting in Ethiopia. We aimed to determine the leading causes of premature mortality and disability using DALYs and describe the relative burden of disease and injuries in Ethiopia.

**Methods:**

We used results from the Global Burden of Diseases, Injuries, and Risk Factors Study 2015 (GBD 2015) for non-fatal disease burden, cause-specific mortality, and all-cause mortality to derive age-standardized DALYs by sex for Ethiopia for each year. We calculated DALYs by summing years of life lost due to premature mortality (YLLs) and years lived with disability (YLDs) for each age group and sex. Causes of death by age, sex, and year were measured mainly using Causes of Death Ensemble modeling. To estimate YLDs, a Bayesian meta-regression method was used. We reported DALY rates per 100,000 for communicable, maternal, neonatal, and nutritional (CMNN) disorders, non-communicable diseases, and injuries, with 95% uncertainty intervals (UI) for Ethiopia.

**Results:**

Non-communicable diseases caused 23,118.1 (95% UI, 17,124.4–30,579.6), CMNN disorders resulted in 20,200.7 (95% UI, 16,532.2–24,917.9), and injuries caused 3781 (95% UI, 2642.9–5500.6) age-standardized DALYs per 100,000 in Ethiopia in 2015. Lower respiratory infections, diarrheal diseases, and tuberculosis were the top three leading causes of DALYs in 2015, accounting for 2998 (95% UI, 2173.7–4029), 2592.5 (95% UI, 1850.7–3495.1), and 2562.9 (95% UI, 1466.1–4220.7) DALYs per 100,000, respectively. Ischemic heart disease and cerebrovascular disease were the fourth and fifth leading causes of age-standardized DALYs, with rates of 2535.7 (95% UI, 1603.7–3843.2) and 2159.9 (95% UI, 1369.7–3216.3) per 100,000, respectively. The following causes showed a reduction of 60% or more over the last 25 years: lower respiratory infections, diarrheal diseases, tuberculosis, neonatal encephalopathy, preterm birth complications, meningitis, malaria, protein-energy malnutrition, iron-deficiency anemia, measles, war and legal intervention, and maternal hemorrhage.

**Conclusions:**

Ethiopia has been successful in reducing age-standardized DALYs related to most communicable, maternal, neonatal, and nutritional deficiency diseases in the last 25 years, causing a major ranking shift to types of non-communicable disease. Lower respiratory infections, diarrheal disease, and tuberculosis continue to be leading causes of premature death, despite major declines in burden. Non-communicable diseases also showed reductions as premature mortality declined; however, disability outcomes for these causes did not show declines. Recently developed non-communicable disease strategies may need to be amended to focus on cardiovascular diseases, cancer, diabetes, and major depressive disorders. Increasing trends of disabilities due to neonatal encephalopathy, preterm birth complications, and neonatal disorders should be emphasized in the national newborn survival strategy. Generating quality data should be a priority through the development of new initiatives such as vital events registration, surveillance programs, and surveys to address gaps in data. Measuring disease burden at subnational regional state levels and identifying variations with urban and rural population health should be conducted to support health policy in Ethiopia.

## Background

Like many other countries in sub-Saharan Africa, Ethiopia is a country of diverse cultures, traditions, and histories [1]. In spite of its ancient civilizations, Ethiopia is still one of the least developed countries, with low development indicators; according to The World Bank report, Ethiopia’s GNI was 590 USD per capita in 2015 [2, 3]. Following its economic progress in the last decades, Ethiopia anticipates joining the category of lower-middle-income countries by 2025, and upper-middle-income countries by 2035 [4]. The pyramidal age structure of the population has remained predominately young, with 44.9% under the age of 15 years, and over half (52%) of the population between the ages of 15–65 years; 3% of the population was over the age of 65 years [5]. The total population for Ethiopia in 2015 was estimated at 90 million [2]. The average fertility trend in recent years has shown significant decline from the 2000 level of 5.5 births to 4.6 births per woman in 2015, but is still nearly twice the world average [6, 7].

In 2015, the Ethiopian health sector finalized its 20-year National Health Sector Development Program (HSDP), focusing on decentralizing and expanding the primary health care system, and encouraging the participation of non-governmental partners [1, 8]. During the 20-year period, the country has shown remarkable progress implementing high-impact health interventions through its flagship community-focused program known as the “Health Extension Program” [8]. For instance, under-5 mortality has decreased by two-thirds, [9] and the maternal mortality ratio decreased from 708 in 1990 to 497.4 in 2013 [10]. Moreover, the estimated average life expectancy at birth increased from 45.5 years in 1990 to 61.7 years in 2013 [11].

Following the HSDP, in 2015, Ethiopia started to implement the Health Sector Transformation Plan (HSTP), a five-year strategic plan to improve the quality and equity of health services and universal health coverage [2].

Obtaining reliable data to measure the progress made with the previous HSDP plan and set benchmarks for HSTP, however, is challenging for Ethiopia. As a result, in setting policy framework and envisioning targets, the country has used different scenarios from lower-middle- and upper-middle-income countries [2]. In order to bridge the gaps in data, new country initiatives have been undertaken, including improving the flow of information in the HSTP and creating a vital events registration system; however, these initiatives may not be fully functional for some time [2, 7, 11, 12]. Currently, the existing data sources on mortality, morbidity, and health risk factors are fragmented and are not systematically analyzed locally, creating difficulties when describing key performance indicators [13]. Meanwhile, estimating the summary measures of health loss is essential in guiding health systems and investments, and setting priorities, especially when considering non-fatal consequences of acute and chronic diseases and injury [14]. Disability-adjusted life years (DALYs) are a summary measure that combines mortality, morbidity, and disability to measure population health [15]. This metric describes the causes and drivers of mortality and morbidity in order to be able to identify successes, unmet needs, and potentially unrecognized emerging threats to population health [16].

The Global Burden of Diseases, Injuries, and Risk Factors Study 2015 (GBD 2015) provides the most comprehensive source of DALY estimates for 195 countries, including Ethiopia [15, 17]. We used GBD 2015 to examine the burden of diseases and injuries from 1990 to 2015 in Ethiopia. We have demonstrated the ranks of causes of age-standardized DALYs and the shift in ranks of different causes and described the relative burden of communicable, maternal, neonatal, and nutritional disorders (CMNN), non-communicable diseases, and injuries. The findings will help measure the performances of HSDP as well as set benchmarks and track HSTP progress in Ethiopia.

## Methods

### Data sources

GBD 2015 data were used to analyze DALYs for Ethiopia. GBD uses all available data sources, meeting quality criteria and rigorous analysis to estimate DALY rates for 195 countries [16]. About 171 site-years of cause of death data sources were used for Ethiopia to provide national estimates, of which 70 were nationally representative and 52 represented subnational locations in Ethiopia. About 102 site-year data sources included sibling history data, and 63 site-year data sources used verbal autopsy data collection techniques [17]. For non-fatal health outcome estimation, 489 site-year data sources were used to generate national estimates for Ethiopia. National representativeness was reported for 87 site-year non-fatal health outcome data sources, and 339 site-year data sources have unknown representativeness. Data were relatively scarce for Ethiopia. In this case, GBD uses two more approaches to estimate cause of death: identifying covariates for each cause and borrowing strength from region and super-region in the modeling process [18]. Approximately 881 site-year covariate data sources were used, from which 343 site-years of data were nationally representative; the remaining 538 were reported unknown. The full details of data sources can be found in the supplementary appendix [15].

#### Modeling

Years of life lost due to premature mortality (YLLs) and years lived with disability (YLDs) were used to calculate DALYs for the complete time-series 1990 to 2015 [16]. The GBD approach to estimate YLLs and YLDs has been described in previous GBD publications [19, 20]. YLLs were computed by multiplying numbers of deaths from each cause, in each age group, by the reference life expectancy in order to measure the gap between mortality rates and the reference life expectancy [21]. YLDs were computed by multiplying the prevalence of the condition with its corresponding disability weight [22]. DALYs are the sum of YLLs and YLDs [23, 24].

Causes of death by age, sex, and year were measured primarily by using cause of death ensemble modeling (CODEm) [11]. A detailed description of CODEm is reported in previous GBD publications [11, 23]. In brief, CODEm tests a wide range of models, such as mixed effects linear models and spatiotemporal Gaussian process regression (ST-GPR) models, and constructs an ensemble model based on the performance of the different models [23]. Out-of-sample predictive validity tests were used to select the ensemble model to estimate morality rates [23].

To estimate YLDs, GBD 2015 used a Bayesian meta-regression method, DisMod-MR 2.1. It was designed to address statistical challenges in the estimation of non-fatal health outcomes, and for the synthesis of sparse and heterogeneous epidemiological data. The sequence of estimation occurs at five levels: global, super-region, region, country, and, where applicable, subnational locations. At each level of the cascade, the DisMod-MR 2.1 computational engine enforces consistency between all disease parameters [20]. For some of the diseases, natural history models, geospatial models, back-calculation models, or registration completeness models were used. Disability weights for 2337 sequelae of the 310 diseases and injuries were used to capture the severity of health loss. YLDs by cause at age, sex, country, and year levels were adjusted for co-morbidity with simulation methods [19, 24].

### Interpretation of the results

We followed GBD categorization of diseases and injuries to present and interpret the results. GBD uses a hierarchy of causes that organizes 306 diseases and injuries into four levels of categories [15]. The first level shows three broad categories: communicable, maternal, neonatal, and nutritional (CMNN) disorders; non-communicable diseases (NCDs); and injuries. Level 2 has 21 mutually exclusive and collectively exhaustive categories; Levels 3 and 4 have 164 and 255 categories, respectively [15]. We present the results in terms of age-standardized DALY, YLD, and YLL rates with 95% uncertainty intervals (UI) [11, 14]. We report positive and negative percentages to show changes from 1990 to 2015.

## Results

### Age-standardized disability-adjusted life years (DALYs)

**Fig. 1:**
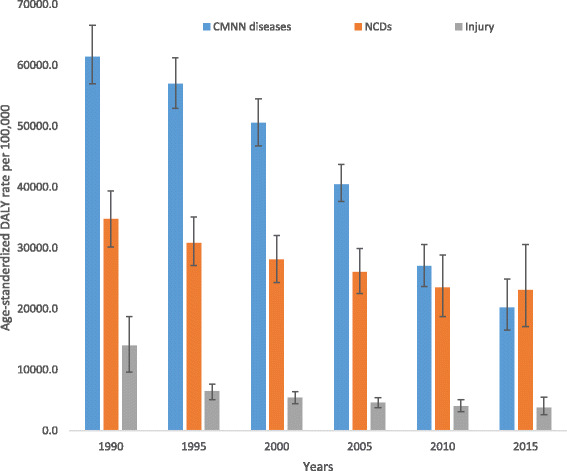
Levels and trends of age-standardized DALYs per 100,000 people by major causes for both sexes and all age groups with 95% UI in Ethiopia, 1990–2015

The disease burden measured in DALYs for CMNN diseases, non-communicable diseases, and injury categories from 1990 to 2015 for both sexes and all ages in Ethiopia is shown in Table 1 and Fig. 1.

**Table 1:** Age-standardized YLLs, YLDs, and DALYs rate per 100,000 peoples for both sexes and all age groups with broad GBD categories in Ethiopia, 1990–2015

Disease and injuries	Age-standardized YLL rates			Age-standardized YLD rates			Age-standardized DALY rates		
	1990	2015	% change	1990	2015	% change	1990	2015	% change
HIV/AIDS and tuberculosis	9005 (6606.5–11,606.6)	4240.7 (2936.7–5927.9)	−53%	229.0 (146.8–347.5)	172.8 (121–233.1)	−25%	9234 (6801.7–11,814.4)	4413.5 (3092.7–6109.8)	−52%
Diarrhea/LRI/others	30,626 (25,637–38,380)	7589 (5799.7–9935.2)	−75%	468.3 (332.9–630.7)	364.5 (260.7–487.2)	−22%	31,094.4 (26,146.9–38,803.6)	7953.5 (6112.7–10,309.1)	−74%
NTDs and malaria	3635.1 (17,34.2–6477.5)	281.8 (169.3–449.7)	−92%	1143.8 (713.7–1860.9)	1055.6 (576–2097.5)	−8%	4778.9 (2785.6–7790.2)	1337.4 (838.1–2456)	−72%
Maternal disorders	2498.2 (1880.6–3299.4)	785.4 (330.8–1742.1)	−69%	164 (112.6–224.1)	45.3 (31.6–62)	−72%	2662.2 (2046.1–3452.5)	830.7 (377.6–1786.5)	−69%
Neonatal disorders	6710.6 (5734.9–7637.5)	3159.7 (2727–3595.2)	−53%	106.1 (63.7–168.3)	137 (85.7–221)	29%	6816.7 (5837.5–7739.7)	3296.7 (2873.9–3735.8)	−52%
Nutritional deficiencies	4098.7 (2017.2–6922.2)	983.4 (577.6–1540.4)	−76%	910.6 (606.5–1311.6)	402.3 (265.4–577.6)	−56%	5009.3 (2883.8–7945.9)	1385.7 (952.6–1968)	−72%
Other CMNN diseases	1714.9 (954.5–2707.4)	910.6 (557.1–1375.8)	−47%	92.9 (60.2–137.4)	72.7 (46.2–118.3)	−22%	1807.8 (1046.2–2813.7)	983.3 (628.1–1460.4)	−46%
Neoplasms	4584.7 (3828.6–5443.7)	3106.9 (1778.5–5136)	−32%	92 (64.6–124.9)	85.2 (46.5–144.7)	−7%	4676.7 (3903.5–5539.1)	3192.1 (1857.2–5221.3)	−32%
Cardiovascular diseases	11,073.6 (9393.1–12,898.8)	5930.7 (3686.2–8993.2)	−46%	484.4 (346.3–656)	527.2 (374.9–703.3)	9%	11,558.1 (9798.5–1,3394.3)	6457.9 (4207.6–9509.3)	−44%
Chronic respiratory diseases	1727.1 (1437.7–2049.1)	751.5 (467.3–1144.6)	−56%	494.8 (372.4–637.8)	381.9 (286–484.2)	−23%	2221.8 (1920.5–2583)	1133.5 (844.3–1515.1)	−49%
Cirrhosis	1764 (1390.1–2135)	802.7 (462–1306.9)	−54%	7.8 (5.5–10.7)	7.2 (5–9.9)	−8%	1771.9 (1398.1–2143.4)	810 (468.8–1315.3)	−54%
Digestive diseases	2126.6 (1740–2632.9)	887.2 (540.7–1375.3)	−58%	327.3 (218–479.9)	207.5 (139–294)	−37%	2453.9 (2026.4–3014.5)	1094.7 (732.9–1587.6)	−55%
Neurological disorders	777.3 (597.1–986.6)	497.5 (318.9–750.6)	−36%	636.3 (436.4–860.9)	621.7 (420.7–854.4)	−2%	1413.7 (1129–1714.8)	1119.2 (837.4–1433.1)	−21%
Mental and substance use disorders	163.6 (118.7–226.8)	86.1 (44–163.7)	−47%	2166.6 (1576.2–2814.2)	2137.5 (1546.4–2791.4)	−1%	2330.2 (1734.7–2995)	2223.5 (1633–2888)	−5%
Diabetes, urogenital, blood, and endocrine diseases	2417.7 (1975.9–2958.3)	1437.3 (892.2–2226.4)	−41%	766.8 (548.1–1020)	755.1 (543.2–994.9)	−2%	3184.5 (2648.4–3787.5)	2192.4 (1584.1–3000.2)	−31%
Musculoskeletal disorders	28.3 (21.4–35.9)	22.4 (11–43)	−21%	1396 (1006.4–1868.3)	1449.3 (1046.4–1943.3)	4%	1424.3 (1040.5–1892)	1471.8 (1069–1974.5)	3%
Other non-communicable diseases	1619.6 (989.6–2416.1)	1239.5 (725.4–1632.1)	−23%	2135.7 (1485.4–3018.7)	2183.6 (1514–3074.7)	2%	3755.3 (2865.9–4868.3)	3423.1 (2615.7–4401.9)	−9%
Transport injuries	1641.4 (1185.5–2104)	782 (485.4–1270.8)	−52%	41.7 (29.9–55.5)	36.3 (25.7–48.3)	−13%	1683.0 (1227.4–2149.4)	818.3 (520.2–1308.8)	−51%
Unintentional injuries	3776.6 (2653.1–4942.7)	1517.5 (1019–2248.3)	−60%	686 (493.2––916.2)	557.8 (402.6––742.1)	−19%	4462.6 (3308.9–5690.8)	2075.3 (1530.5–2833.7)	−53%
Self-harm and violence	1319.2 (944.9–2150.2)	771.8 (424–1403.1)	−41%	16 (11.6–21.3)	14 (9.9–18.4)	−13%	1335.3 (960.7–2163.2)	785.8 (438.2–1413.9)	−41%
War and disaster	6153.3 (2258.8–10,730.9)	20.1 (6.1–36.9)	−100%	340 (158.1–661.6)	81.5 (48.8–131.4)	−76%	6493.3 (2672.2–11,093.3)	101.6 (63.7–153.7)	−98%

**Fig. 2:**
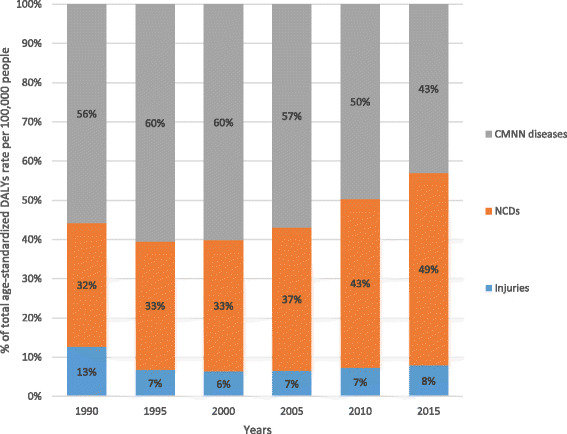
Percentage contribution to total age-standardized DALYs per 100,000 people for major causes for both sexes and all age groups in Ethiopia, 1990–2015

**Fig. 3:**
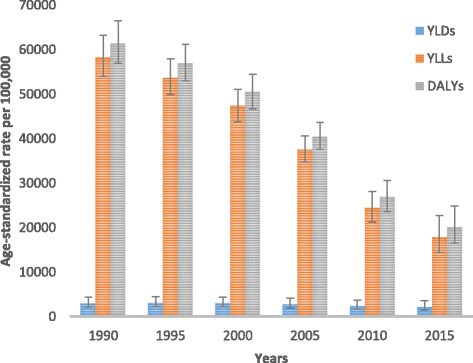
Levels of age-standardized YLLs, YLDs, and DALYs per 100,000 people due to communicable, maternal, neonatal, and nutritional diseases for both sexes and all age groups in Ethiopia, 1990–2015

**Fig. 4:**
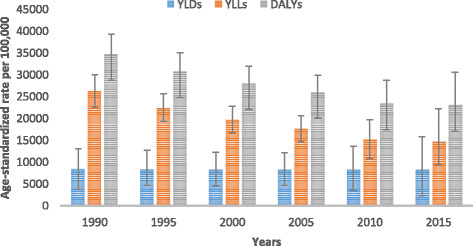
Levels of age-standardized YLLs, YLDs, and DALYs per 100,000 people due to non-communicable diseases for both sexes and all age groups in Ethiopia, 1990–2015

**Fig. 5:**
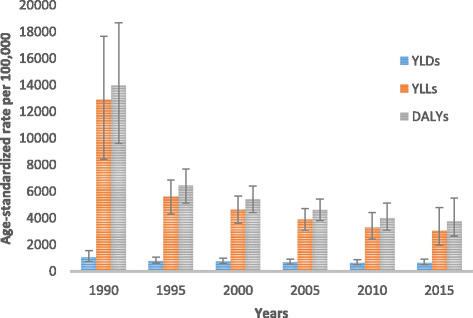
Levels of age-standardized YLLs, YLDs and DALYs per 100,000 people due to injuries for both sexes and all age groups in Ethiopia, 1990–2015

All-cause age-standardized DALYs per 100,000 were 47,099.8 (95% UI, 36,546.7–60,314.2) in 2015. Of this total, CMNN disorders caused 20,200.7 (95% UI, 16,532.2–24,917.9), NCDs caused 23,118.1 (95% UI, 17,124.4–30,579.6), and injuries caused 3781 (95% UI, 2642.9–5500.6) of the DALYs (Fig. 1). The percentage contribution of CMNN diseases to total age-standardized DALYs declined from 56% in 1990 to 43% in 2015, while that of NCDs increased from 32% to 49% during the same period (Fig. 2). Age-standardized DALYs per 100,000 from all-cause mortality declined from 1990 to 2015: rates of DALYs caused by CMNN diseases declined from 61,403.3 to 20,200.7, DALY rates from NCDs declined from 34,790.3 to 20,200.7, and DALY rates due to injuries from 13,974.2 to 3781.0 (Fig. 1). The decreasing trend of DALY rates over the years for the three broad categories continued with respect to age-standardized YLL rates, but the same was not true for YLDs (Figs. 3, 4, and 5). Between 1990 and 2015, the contribution of age-standardized YLD rates respective to DALY rates was higher for NCDs compared to YLD rates due to CMNN diseases or injuries. In 2015, YLD rates contributed 36% of the total DALYs for NCDs, and the rate was constant over the years.

Diarrhea, lower respiratory infections, and other infections combined caused 7953.5 (95% UI, 6112.7–10,309.1) age-standardized DALYs per 100,000 in 2015. The age-standardized DALY rate for HIV/AIDS and tuberculosis combined was 4413.5 (95% UI, 3092.7–6109.8), and neonatal disorders caused 3296.7 (95% UI, 2873.9–3735.8) DALYs per 100,000 (Table 1).

Cardiovascular diseases and neoplasms caused 6457.9 (95% UI, 4207.6–9509.3) and 3192.1 (95% UI, 1857.2–5221.3) DALYs per 100,000, respectively (Table 1). Unintentional and transport injuries caused 1214.6 (95% UI, 1017.4–1418) and 818.3 (95% UI, 520.2–1308.8) DALYs per 100,000, respectively (Table 1).

From 1990 to 2015, all causes of DALYs except musculoskeletal disorders showed declines, with a major reduction of age-standardized DALYs occurring within CMNN group causes (Table 1). Age-standardized DALY rates from diarrhea, lower respiratory infections, and other infections collectively declined by 74%, the YLL rate declined by 75%, and the YLD rate declined by 22% during the 25-year period.

The combined age-standardized DALY rate from HIV/AIDS and tuberculosis declined by 52% (Table 1), and the reduction followed the YLL rate pattern of 53% more closely than the YLD rate decline of 25%. The age-standardized DALYs per 100,000 from cardiovascular diseases and neoplasms showed 44% and 32% reductions in rate between 1990 and 2015, respectively. The reduction in cardiovascular diseases and neoplasms was mainly from YLL rates, whereas the YLD rates showed only a 7% reduction for neoplasms and a 9% increase for cardiovascular diseases. Unintentional injury declined by 53% and transport injury by 51% (Table 1). The YLL rate reduction was high for transport and unintentional injuries compared with their YLD rates.

**Fig. 6:**
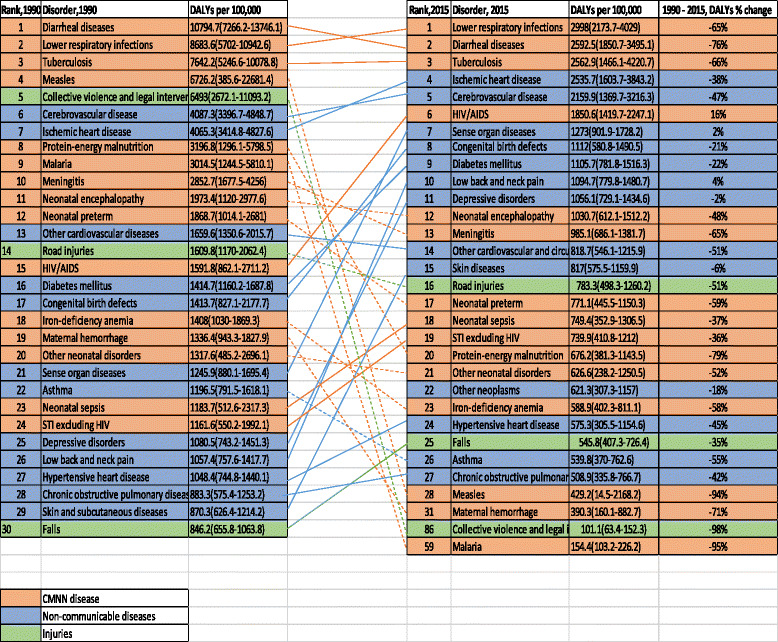
Ranks of causes of age-standardized DALYs per 100,000 for both sexes and all age groups in Ethiopia, 1990–2015

Ranks of specific diseases with their age-standardized DALY rates and percentage changes between 1990 and 2015 are shown in Fig. 6. The top 20 leading causes of age-standardized DALYs accounted for 59% of the total DALYs in Ethiopia. Lower respiratory infections, diarrheal diseases, and tuberculosis were the top three leading causes of DALYs, accounting for 2998 (95% UI, 2173.7–4029), 2592.5 (95% UI, 1850.7–3495.1), and 2562.9 (95% UI, 1466.1–4220.7) per 100,000, respectively (Fig. 6). Ischemic heart disease and cerebrovascular disease were the fourth and fifth leading causes of age-standardized DALY rates, with 2535.7 (95% UI, 1603.7–3843.2) and 2159.9 (95% UI, 1369.7–3216.3), respectively. HIV/AIDS was the sixth leading cause of DALYs, followed by diabetes mellitus and major depressive disorders.

**Fig. 7:**
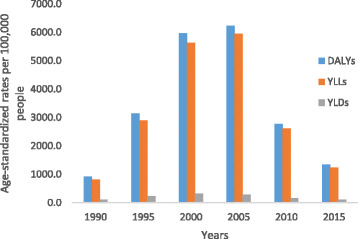
Levels of age-standardized YLLs, YLDs, and DALYs per 100,000 people due to HIV/AIDS for both sexes and all age groups in Ethiopia, 1990–2015

**Fig. 8:**
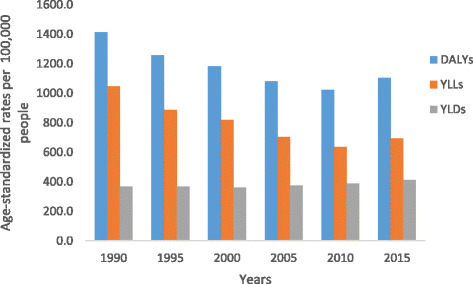
Levels of age-standardized YLLs, YLDs, and DALYs per 100,000 people due to diabetes for both sexes and all age groups in Ethiopia, 1990–2015

**Fig. 9:**
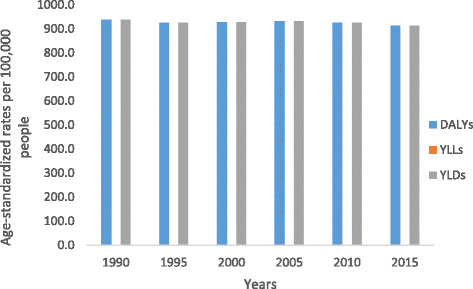
Levels of age-standardized YLLs, YLDs, and DALYs per 100,000 people due to major depression for both sexes and all age groups in Ethiopia, 1990–2015

Between 1990 and 2015, there was shifting in ranks of disease types as certain non-communicable diseases joined the top 20 causes, replacing most CMNN causes. For example, measles, protein-energy malnutrition, and malaria were among the top causes of age-standardized DALYs in 1990 but not in 2015, as diabetes, low back pain, and major depressive disorders ranked in the top 10 in 2015. Between 2005 and 2015, HIV/AIDS burden decreased by 78% (Figs. 7, 8 and 9).

The top 30 leading causes of age-standardized DALY rates showed reductions from 1990 to 2015 except low back pain and sense organ diseases, as shown in Fig. 6. Significantly larger reductions occurred in the first three leading causes of DALYs: lower respiratory infections decreased by 65%, diarrheal diseases decreased by 76%, and tuberculosis decreased by 66% (Fig. 6). Ischemic heart disease declined by 38% and cerebrovascular disease by 47% (Fig. 6). As presented in Fig. 6, most non-communicable diseases in the 20 leading causes of DALYs generally showed lower percentage declines compared to CMNN diseases between 1990 and 2015. The following causes showed reductions of 60% or more: lower respiratory infections, diarrheal diseases, tuberculosis, neonatal encephalopathy, preterm birth complications, meningitis, malaria, protein-energy malnutrition, iron-deficiency anemia, measles, war and legal intervention, and maternal hemorrhage (Fig. 6).

The contribution of age-standardized YLL and YLD rates to DALYs varies by diseases types over time. For example, the reduction in age-standardized DALYs from ischemic heart disease from 1990 to 2015 followed the YLL rate, declining by 39%, while the YLD rate increased by 16%. The age-standardized DALY rate due to cerebrovascular disease followed the same pattern as its YLL rate; however, the YLD rate increased by 21%. The same is true for diabetes and congenital birth defects (Table 2). Similarly, the age-standardized DALY rate for neonatal disorders decreased, following the YLL rate pattern, as the YLD rate increased. Neonatal encephalopathy showed a 50% decrease in the age-standardized YLL rate and a 30% increase in the YLD rate. Similarly, neonatal preterm birth complications showed a 60% decrease in the DALY rate, while the YLD rate increased by 38% between 1990 and 2015 (Table 2). The rate of decline in DALYs per 100,000 was mainly due to a decrease in YLLs rather than YLDs, similar to the leading causes, lower respiratory infections, diarrheal diseases, and tuberculosis.

**Table 2:** Age-standardized YLLs and YLDs rate per 100,000 peoples for both sexes and all age groups for 20 leading causes of DALYs in Ethiopia in 2015. *excluding HIV/AIDS and other causes

Disease and injuries	Age-standardized YLL rates			Age-standardized YLD rates		
	1990	2015	% change	1990	2015	% change
Lower respiratory infections	8668.2 (5688.1–10,930.2)	2987.2 (2165.5–4017.9)	−66%	15.4 (10.4–21.7)	10.8 (7.3–15.3)	−30%
Diarrheal diseases	10,530.6 (7001.5–13,496.6)	2405.2 (1676.8–3309.1)	−77%	264 (181.9–361.6)	187.3 (129.3–257)	−29%
Tuberculosis	7642.2 (5246.6–10,078.8)	2502.3 (1410.4–4151.9)	−67%	109.8 (74–147.8)	60.6 (40.8–83.1)	−45%
Ischemic heart disease	3931.6 (3286.5–4694.5)	2380.3 (1446.5–3680)	−39%	133.7 (88.7–186.9)	155.4 (103.2–214.9)	16%
Cerebrovascular disease	3967 (3277.3–4729.8)	2014.9 (1224.4–3086.3)	−49%	120.3 (83.4–159.9)	145 (101.6–193.5)	21%
Sense organ diseases	0	0	0	1246.0 (880.6–1694.6)	1273.0 (902.4–1725.8)	2%
Congenital birth defects	1353.2 (768.6–2121.7)	1035.2 (508.6–1410.4)	−23%	60.5 (32.1–108.5)	76.7 (45.2–127.8)	27%
Diabetes mellitus	1047.8 (840.2–1295.7)	692.4 (415–1096.1)	−34%	366.9 (252.6–500.3)	413.3 (279.5–558.7)	13%
Low back and neck pain	0	0	0	722.0 (513.9–992.1)	756.1 (536.3–1044.8)	5%
Depressive disorders	0	0	0	937.1 (629.4–1274.3)	912.3 (614.4–1254.6)	−3%
Neonatal encephalopathy	1911.4 (1052.5–2917.3)	950.1 (537–1428.4)	−50%	62 (35.1–98.1)	80.6 (46.7–127.5)	30%
Meningitis	2779.7 (1603.5–4178.8)	918.3 (613.3–1317.3)	−67%	73.0 (50.9–95.9)	66.9 (47.0–88.4)	−8%
Other cardiovascular diseases	1529.7 (1208.8–1877.3)	676.5 (417.7–1066.2)	−56%	130.0 (75.5–207.9)	142.2 (84.1–222.4)	9%
Skin diseases	0	0	0	639.8 (415.4–952.2)	643.9 (417.3–962.7)	1%
Road injuries	1575.1 (1138.4–2027.6)	752.8 (468.9–1229.9)	−52%	34.7 (24.5–46.7)	30.5 (21.6–40.9)	−12%
Neonatal preterm	1853.8 (999.7–2666.8)	750.3 (426.5–1127.9)	−60%	15.0 (10.4–21.8)	20.7 (14.5–27.9)	38%
Neonatal sepsis	1183.4 (512.4–2316.9)	749.1 (352.7–1306.2)	−37%	0.3 (0.2–0.6)	0.3 (0.1–0.5)	−18%
STI excluding HIV	1110.8 (500.0–1942.3)	693.0 (365.0–1170.4)	−38%	50.8 (32.9–78.1)	46.9 (30.0–72.7)	−8%
Protein-energy malnutrition	3123.1 (1223.1–5742.4)	622.7 (324.4–1094.6)	−80%	73.7 (35.8–132.1)	53.5 (27.5–87.5)	−28

## Discussion

Comparing point estimates for Ethiopia in 2015, age-standardized DALY rates caused by non-communicable diseases were higher than those due to CMNN diseases. Between 1990 and 2015 there was a larger decrease in the DALY rate from CMNN diseases than from non-communicable diseases between 1990 and 2015. The higher reduction of DALY rates due to CMNN disorders occurred following a major decline in premature mortality rates, as measured in age-standardized YLL rates; however, the reduction of the age-standardized YLD rate was minimal. Although the reduction in age-standardized YLL rates caused non-communicable disease DALYs to decline, the YLD rates did not decline proportionally. Overall, the YLD rate contribution to total DALYs was high, resulting in minimal reduction in DALYs caused by non-communicable diseases over the 25-year period.

There are two possible explanations for the current burden of non-communicable diseases in Ethiopia. The first possibility is that the burden from non-communicable diseases has truly increased and become highly prevalent in urban settings due to behavior and lifestyle changes. The other, however, postulates that non-communicable diseases were existing problems and became more visible with a greater reduction in CMNN diseases, increased population size, and/or aging. The findings in this paper could help to elucidate both possibilities; the absolute number of DALYs due to non-communicable diseases has increased and DALYs due to CMNN diseases decreased; age-standardized DALYs, after controlling for population growth and aging, decreased over the 25 years in both categories.

Age-standardized DALYs due to non-communicable diseases declined slowly compared to CMNN diseases; the YLD rate of non-communicable diseases was stable over the last 25 years in Ethiopia. This could support the fact that non-communicable diseases were not among the country’s priorities as national strategies and action plans were not in place until 2010 [25]. Moreover, the age-standardized DALY rate reduction for CMNN diseases was mainly driven by decreases in lower respiratory infections, diarrheal diseases, tuberculosis, measles, malaria, protein-energy malnutrition, and others. These findings support the direction and investment of the health sector development plan in achieving the Millennium Development Goals (MDGs) and other CMNN disease targets for the last 25 years [1]. The plan appears to be successful in reducing the burden of most CMNN diseases and may address certain non-communicable diseases with infectious disease origin, such as rheumatic heart disease.

The findings of this study supported the claim made in other studies about the double burden of CMNN disease and non-communicable diseases occurring in the process of epidemiologic transition in Ethiopia [26–29]. However, there could be variation across different geographies and populations within the country. A rural-based study showed that CMNN diseases constituted 72% of the total DALYs and NCDs contributed 24% [30]. It is challenging to compare this estimate with GBD findings as there are methodological, study population, and other major differences.

More specifically, lower respiratory infections, diarrheal diseases, and tuberculosis have continued to be the three leading causes of age-standardized DALY rates in 2015 as in 1990, although they showed remarkable decline over the last 25 years. In addition, the following leading diseases showed a reduction of 60% or more between 1990 and 2015: meningitis, malaria, protein-energy malnutrition, iron-deficiency anemia, measles, and maternal hemorrhage. This reduction in the leading causes of DALYs occurred following a reduction in premature mortality, as the YLD rate contribution to the total DALY rate was less.

The level of DALYs due to HIV/AIDS increased until 2005 and then decreased until 2015. This pattern was highly influenced by the disease’s level and ability to cause premature mortality over the years. In 2000, disability caused by HIV/AIDS, as measured by the age-standardized YLD rate, peaked at 323 per 100,000 people and declined to 100 per 100,000 in 2015. However, this result may not be uniform across the different geographic and sociodemographic population groups in Ethiopia. This requires improving equity and access to antiretroviral therapy (ART) [31].

In contrast, age-standardized YLD rates caused by neonatal disorders have increased over the years. For example, rates of YLDs caused by neonatal encephalopathy increased by 30%, and those caused by neonatal preterm birth complications increased by 38% over the last 25 years. Although Ethiopia has achieved Millennium Development Goal 4 (MDG 4) of reducing child mortality, reports showed that the decline in neonatal mortality was not as impressive as that of infant and child mortality [32]. This implies that current national newborn survival strategies, in the continuum of care approach, need to prioritize the increasing trend of disabilities due to neonatal disorders in addition to premature mortality [32].

Except for the three leading causes of DALYs, the other CMNN disease types that were leading causes in the 1990s have been replaced by several non-communicable diseases in 2015. The fourth and fifth ranked causes of DALYs were ischemic heart disease and cerebrovascular diseases. Both diseases caused increasing rates of disability over the years. The same is true for diabetes and congenital birth defects, as age-standardized YLD rates increased between 1990 and 2015.

On the other hand, DALYs caused by major depressive disorder were only from disability outcomes as measured in age-standardized YLD rate, and that showed precisely no change over the years. This finding might reflect the fact that mental health was not in the priority national health agenda until national mental health strategies were developed in 2012 [33]. The integrated mental health approach, which integrates mental health care within existing primary care, needs to address the high level of disabilities caused by major depressive disorder.

### Implications of the findings for policies and practices

Primarily, the findings are useful for highlighting the performance of the previous health sector development plan and the MDGs, and to benchmark the health sector transformation plan and the Sustainable Development Goals at the country level [2, 34].

### Improvements in common infectious diseases

Lower respiratory infections, diarrhea, and other common infectious diseases are still Ethiopia’s largest drivers of premature death and disability as measured in DALY rates; however, they are on the decline for all ages and both sexes. The age-standardized DALY rate from these diseases declined 74% between 1990 and 2015 (from 31,094 DALYs per 100,000 in 1990 to 7953 in 2015). This decline has contributed to Ethiopia’s ability to achieve MDG 4, reducing under-5 mortality by two-thirds [13]. Many different types of interventions might have impacted this decline: increased health service coverage and utilization for early detection and treatment, community-based interventions under the flagship of health extension workers, integrated management of childhood illnesses, and increased childhood immunizations [35]. In spite of the ease with which these diseases are preventable and treatable, the high rate of premature deaths and disabilities due to common infectious diseases is still unacceptably high in Ethiopia. There is a need to scale up existing high-impact interventions, especially in populations whose coverage lags behind the target in skilled birth attendance and early postnatal care, treatment interventions including oral rehydration therapy and zinc, and antibiotics for pneumonia. There is also a need to increase the coverage and utilization of newly introduced interventions in the country, including community management of childhood illness (pneumonia, diarrhea, and malnutrition) neonatal sepsis, and community-based nutrition, Interventions that improve access to *Haemophilus influenzae* type b, pneumococcal, and rotavirus vaccines, among others, could be implemented to decrease the burden of these common infectious diseases in Ethiopia [32].

### Progress in combating HIV/AIDS, tuberculosis, and malaria

Premature mortality and disability rates from malaria fell drastically between 1990 and 2015, dropping 91% (from 1764 age-standardized DALYs per 100,000 in 1990 to 154 in 2015); however, HIV/AIDS and tuberculosis are still Ethiopia’s leading drivers of premature mortality and disability. Age-standardized DALY rates from HIV/AIDS fell by 78% between 2005 and 2015 (from 8396 DALYs per 100,000 in 2005 to 1850 in 2015), and the decline followed the premature mortality rate trends. Country progress reports indicate that HIV incidence has also decreased by 90% since 2005 [2].

The expansion of free antiretroviral treatment (ART) since 2005 may have contributed to the reduction in premature mortality rates in Ethiopia [36]. Some studies also show that the decline in mortality among individuals living with HIV/AIDS in Ethiopia is consistent with ART uptake [36, 37]. However, the contribution of the age-standardized disability rate (112 YLDs per 100,000) to total DALYs was relatively small over the years compared to premature mortality rates, and fell by 65% between 2005 and 2015. On the other hand, ART coverage in adults (68%) and children (23%) is still very low in Ethiopia, which contributes to premature mortality in vulnerable populations [38]. Thus, improving universal access to ART, retention of enrolled clients in treatment services, and adherence to treatment are necessary to reduce premature mortality from HIV/AIDS. However, there is a need to seek strategies for the sustainability of ART services in terms of funding for Ethiopia. Prevention activities need to continue with a focus on geographic “hot spots” and most at-risk populations.

Although the total age-standardized DALY rate for tuberculosis (TB) decreased 65% between 1990 and 2015, TB caused higher premature death and disability rates in 2015 (2563 age-standardized DALYs rate per 100,000 people). According to the 2014 WHO report, the prevalence and incidence of all forms of tuberculosis were 211 and 224 per 100,000 people, respectively [39]. Ethiopia is one of the countries that has a high burden of HIV/tuberculosis co-infection as well as multidrug-resistant tuberculosis. About 13% of all new tuberculosis cases were HIV co-infected, and of all tuberculosis patients with known HIV status, about 11% were HIV co-infected [39]. According to the national tuberculosis drug resistance surveillance report, 2.3% of new tuberculosis cases and 17.8% of previously treated tuberculosis cases were estimated to be multidrug-resistant [39]. There is a need to address tuberculosis/HIV co-infection and multidrug-resistant tuberculosis in order to improve the premature mortality rate from tuberculosis in Ethiopia. This can be done by increasing tuberculosis case detection rate (74%), treatment success rate (83%), and tuberculosis cure rate (67%) [2].

### Increasing importance of non-communicable diseases

In 2015 cardiovascular diseases, cancer, diabetes, and mental and substance use disorders accounted for 30% of the total disease burden in the country as measured in age-standardized DALY rates. Cardiovascular diseases, including ischemic heart disease, stroke, and hypertensive heart disease, were Ethiopia’s second leading cause of premature death and disability (6458 age-standardized DALYs per 100,000). Cancer was the sixth leading cause of premature death and disability in 2015 (causing 3192 age-standardized DALYs per 100,000). Cervical, liver, breast, and esophageal cancers are some of the leading drivers of poor health from cancer. Mental and substance use disorders, including major depression, were the seventh leading cause of premature death and disability (2224 DALYs per 100,000). In 2015, diabetes was the ninth leading cause of premature death and disability (causing 1106 DALYs per 100,000). A large percentage of these non-communicable diseases are preventable through the reduction of the four main shared behavioral risk factors, all of which are widely prevalent in Ethiopia: unhealthy diet, the harmful use of alcohol, tobacco use, and lack of physical activity [2]. Up to 80% of heart disease, stroke, and type 2 diabetes, and about 40% of cancers could be prevented by controlling these risk factors [40]. These behavioral risk factors lead to more formidable metabolic risk factors, including high blood pressure, high blood glucose, high blood lipids, overweight, and obesity [32]. High prevalence of overweight, obesity, and associated high blood pressure were also reported in urban areas of Ethiopia [29, 41].

In light of high disease burden from cardiovascular disease, cancer, diabetes, mental and substance use disorders in Ethiopia, and their shared behavioral risk factors, prevention and treatment interventions need to be reinforced and put in place at different levels. The impact of these diseases and their risk factors could affect sectors beyond health, and the response requires a multi-sectoral approach. The health, agriculture, trade and industry, education, urban planning, and transport sectors need to be nationally coordinated to provide a multi-sectoral response against the leading non-communicable diseases. Initiatives to establish cancer treatment centers, services for cardiovascular disease, and mental illnesses need to be strengthened [2].

Ethiopia has already ratified the WHO Framework Convention on Tobacco Control (FCTC) and the Protocol to Eliminate Illicit Trade in Tobacco in 2015 [42]; however, there is a need to develop implementation guidelines and regulatory mechanisms that can enforce the convention. Reducing tobacco use by establishing tobacco-free environments, increasing public knowledge of the hazards of tobacco, and continuously increasing taxes on the purchase and sale of tobacco products are recommended interventions in the convention. There is also a need to develop and implement guidelines on alcohol use in Ethiopia.

Health services for cardiovascular diseases, cancers, and diabetes need to be integrated within existing primary health care and extended to secondary and tertiary health facilities. Preventive efforts on cervical cancer screening, hypertension screening, diabetes screening, and HBV vaccination need to be strengthened [2]. There is also a need to involve the private sector and civic society in delivering health services for cardiovascular disease, cancer, diabetes, and their risk factors. Besides, institutional capacity developments and human resource trainings, including pre-service and in-service trainings, need to consider addressing the double burden of common infectious diseases (lower respiratory infections, diarrheal disease), HIV/AIDS and tuberculosis, and cardiovascular disease, cancer, and diabetes. The health disparities between urban and rural populations and between subnational regional states and districts requires further exploration to support public health policy at each level.

### Key investments for Ethiopia to reduce uncertainty

The 95% UI for premature death and disability estimates varies by causes that positively or negatively affect health policy debates and decisions for Ethiopia. The lack of data is reflected by wider uncertainty intervals. For example, 95% uncertainty intervals for estimates of age-standardized DALYs per 100,000 for lower respiratory infection, diarrhea, HIV/AIDS, tuberculosis, and malaria showed relatively better uncertainties. However, uncertainties for cardiovascular disease, 6457.9 (95% UI, 4207.6–9509.3) and cancer, 3192 (95% UI, 1857.2–5221.3), estimates were massive, which could impact prioritization and policy decisions. The uncertainties depend on the availability and accuracy of data sources. Therefore, diseases having wider uncertainty intervals require key investments to generate comprehensive and quality data. This concern has been apparent with most non-communicable diseases.

Moreover, limitations of these estimates are discussed widely and in detail in the published GBD 2015 articles; however we summarized the relevant limitations here focusing on data sources [1, 5, 14, 24, 43]. The standard GBD modeling assists in overcoming issues associated with poor-quality data. First, there was lack of data from Ethiopia, and making estimates for a country over time is challenging with few or no data. There were relatively more covariate data source inputs (881 site-years) than non-fatal health outcomes (489 site-years) or causes of death (171 site-years) in GBD 2015 to estimate burden of disease for Ethiopia.

Almost all data sources for causes of death were verbal autopsy and sibling history studies. Sibling history data were nationally representative but were limited to maternal health. Verbal autopsy data sources were all sub-nationally representative and were used to provide national estimates. In addition, there could be variations within verbal autopsy data sources that could affect uncertainty of national estimates [23]. Addressing and reporting all age groups and all possible causes of death, including non-communicable diseases, would be challenging for verbal autopsy data sources. In the non-fatal outcome data sources, only 87 site-year data sources were nationally representative and 339 site-years were reported unknown, which could affect the uncertainty level of the estimates. Moreover, data accessibility is a critical challenge for which GBD 2015 estimates were forced to use scientific literature that was not comprehensive and detailed in reporting. These sources reported broad age categories, lacked reporting by sex, and may have been influenced by other publication bias. This resulted in exclusion of some data sources or adjustment to a higher cause level. This requires close collaboration using actual data sources to access comprehensive and detailed data for GBD estimates. All these limitations with the different data sources may have affected the uncertainty of the estimates.

Until good-quality vital statistics are attained for Ethiopia, investing in national verbal autopsy studies, such as post-census or post-demographic health surveys, and/or further expansion of demographic surveillance sites, would assist in filling existing data gaps in Ethiopia. There is a need to develop national data-sharing guidelines, a culture of data sharing, and a national data center or data archive in the country. Studies should be released in a timely manner to maximize their use.

Finally, Ethiopia requires high investment and commitment to generate quality cause of death data through Vital Events Registration Agency (VERA), which was established in August 2016 [43]. There is also a need to establish and strengthen surveillance systems and surveys to generate national and subnational non-fatal health outcome data with emphasis on leading non-communicable diseases and their shared risk factors. Estimates did not show the possible heterogeneous figures on diseases and disorders by rural and urban or subnational populations as sociodemographic, lifestyle, and health risk factors, which may vary [2, 15]. Therefore, subnational regional states could benefit from subnational burden of disease estimates in the future.

## Conclusions

Ethiopia has been successful in reducing age-standardized DALYs related to most communicable, maternal, neonatal, and nutritional deficiency diseases for the last 25 years. This has resulted in a shift in the leading causes of DALYs from CMNN disorders to non-communicable diseases between 1990 and 2015. Lower respiratory infections, diarrheal diseases, and tuberculosis were still leading causes of premature death and disability in 2015 despite major reductions. Increasing trends of disabilities due to neonatal encephalopathy, neonatal preterm birth complications, and neonatal disorders should be emphasized in the national newborn survival strategy. Premature mortality caused by non-communicable diseases showed reductions, although disability rates did not decline. Because of this, integrated strategies in the health system and a multi-sectoral response should be enacted. Strategies for non-communicable diseases need to focus on the leading causes, such as cardiovascular diseases, cancer, diabetes, and mental and substance use disorders. Generating quality data through new initiatives such as vital events registration, surveillance programs, and surveys should be a priority. There should also be a data-sharing mechanism to pool multiple data sources together to answer national and subnational health indicators. Moreover, there is a need to measure disease burden at subnational regional state levels and identify variations with urban and rural populations to support health policy in Ethiopia.

## Abbreviations

CODEm: Cause of death ensemble modeling
DHS: Demographic and health surveys
GBD: Global Burden of Disease
HIV/AIDS: Human immunodeficiency virus/acquired immune deficiency syndrome
HSDP: Health Sector Development Program
HSTP: Health Sector Transformation Plan
MDGs: Millennium Development Goals
NCDs: Non-communicable diseases
ST-GPR: Spatiotemporal gaussian process regression
UI: Uncertainty intervals
WHO: World Health Organization
YLL: Years of life lost
